# AtPAP2, a Unique Member of the PAP Family, Functions in the Plasma Membrane

**DOI:** 10.3390/genes9050257

**Published:** 2018-05-17

**Authors:** Qingqing Sun, Jinyu Li, Wenzhen Cheng, Huihong Guo, Xiaomin Liu, Hongbo Gao

**Affiliations:** College of Biological Sciences and Biotechnology, Beijing Forestry University, Beijing 100083, China; sunqq@bjfu.edu.cn (Q.S.); lijinyu2009@hotmail.com (J.L.); chengwenzhen@bjfu.edu.cn (W.C.); guohh@bjfu.edu.cn (H.G.); gaohongbo@bjfu.edu.cn (H.G.)

**Keywords:** purple acid phosphatase, secretory pathway, transmembrane domain, phosphorus nutrition, carbon metabolism, phylogenetic analysis

## Abstract

Purple acid phosphatases (PAPs) play various physiological roles in plants. AtPAP2 was previously shown to localize to both chloroplasts and mitochondria and to modulate carbon metabolism in *Arabidopsis*. Over-expression of AtPAP2 resulted in faster growth and increased biomass in several plant species, indicating its great potential for crop improvement of phosphate use and yield. Here, we studied the localization of AtPAP2 by transient expression in tobacco leaves. The results showed AtPAP2 was localized to the plasma membrane through the secretory pathway, which is different from previous studies. We also found that AtPAP2 had a close relationship with fungal PAP2-like proteins based on phylogenetic analysis. In addition, the C-terminal transmembrane domain conserved in land plants is unique among other AtPAPs except AtPAP9, which is a close homolog of AtPAP2. Taken together, our results provide information for further study of AtPAP2 in understanding its special function in crop improvement.

## 1. Introduction

Phosphorus (P) is one of the macronutrients obtained from the soil by roots and required for plant growth and development. It is a component of sugar phosphates, nucleic acids, nucleotides, coenzymes, phospholipids, phytic acid, etc., which are important in structural integrity and signal transduction, and also has a key role in reactions that involve ATP, an important form of energy storage. Although P is abundant in the earth’s crust, it is usually present in the form of organophosphate which is difficult for plants to use. To obtain high crop yield, inorganic phosphate (Pi) fertilizer is widely used in modern farming to support the growing population, now and in the future. However, over-fertilization not only increases the cost of crop production but also leads to eutrophication of water bodies. The studies of how plants obtain and use P and how to develop crops with improved P utilization efficiency are important for sustainable agriculture.

Organophosphate accounts for up to 30~65% of total P in soil [1]. In the face of this edaphic condition, plants must maintain cellular P homeostasis by employing a range of strategies. For most plants, this generally involves the cessation of primary root growth and enhanced lateral root development, secretion of acid phosphatases, as wells as changes in root-microbiome interactions [2]. Plant purple acid phosphatases (PAPs) constitute the largest group of acid phosphatases (EC 3.1.3.2) which catalyze the hydrolysis of Pi from a broad range of phosphomonoesters and anhydrides under acidic conditions. Pi-starvation induced intracellular PAPs are likely involved in the remobilization and recycling of Pi from intracellular P monoesters and anhydrides of older tissues, whereas extracellular or secreted PAPs are thought to scavenge Pi from organophosphate compounds in the external environment [3].

Plant PAPs are distributed across a wide range of plant species. They play multiple roles during plant growth and development, including the acquisition of Pi [4], embryo development [5], and pathogen defense [6,7,8,9]. These enzymes are generally divided into two groups, specific and non-specific, based on their particular catalyzing substrates, function in the production, transport, and recycling of Pi [3]. Multiple PAP-like isoforms have been identified in the genomes of *Arabidopsis thaliana* [10,11], *Zea mays* [12], tomato [13], soybean [14], potato [15] and in prokaryotic genomes [11].

In *Arabidopsis*, there are 29 *PAP* genes, which have been classified into three distinct phylogenetic groups according to their deduced amino acid sequences [10]. Transcript profiling of the *AtPAP* family revealed that most of them are expressed in all tissues, while some *AtPAP* transcripts accumulate in response to Pi limitation [10,16]. Although the molecular and biochemical properties of a series of plant PAPs have been well documented, their precise physiological functions have not been resolved. To date, only some of the *PAP* genes have been functionally characterized in *Arabidopsis*. There is also a paucity of information regarding the subcellular location of most AtPAPs. The root-secreted AtPAP10/12/26 are involved in extracellular phosphate-scavenging [4,17,18,19,20,21]. AtPAP15, with phytase activity, likely mobilizes phosphorus reserves in plants, particularly during seed and pollen germination [22]. AtPAP5 functions in plant defense responses [8,9]. Several *AtPAPs*, such as *AtPAP23*, are predominantly transcribed in flowers, indicating their roles in flower development [16]. The P-starvation inducible AtPAP25 appears to be a phosphoprotein phosphatase rather than a non-specific scavenger of Pi from extracellular P-monoesters during Pi deprivation [23]. In summary, the *AtPAP* gene family has multiple roles in plant growth and development, thus making it difficult to experimentally assess the contributions of individual genes because of redundancy.

AtPAP2 was previously shown to localize to both chloroplasts and mitochondria and to modulate carbon metabolism in *Arabidopsis* [24,25]. Over-expression of *AtPAP2* resulted in faster plant growth in *Camelina sativa* [26], potato [27] and *Arabidopsis* [24]. Metabolite analysis showed that the *AtPAP2* overexpression lines contained higher levels of sugars and tricarboxylic acid (TCA) metabolites, suggesting that the changed carbon metabolism resulted in faster growth and higher yield [24]. Therefore, the *PAP2* gene has great potential for crop improvement of P use and yield. In contrast to the previous report of the localization of AtPAP2, our bioinformatics analysis suggested that it has an N-terminal signal peptide (SP) which is essential for driving the protein into the endomembrane system in the protein secretion pathway. To determine the real localization of AtPAP2 in the cell, we made constructs of yellow fluorescent protein (YFP) fusion and conducted *Agrobacterium*-mediated transient expression in tobacco leaves using the full-length and SP fusion protein. Furthermore, we analyzed the phylogenetic relationships of AtPAP2.

## 2. Materials and Methods

### 2.1. Bioinformatics Analysis of AtPAP2

Homologous protein sequences of AtPAP2 in *A. thaliana* and other species were searched with The Arabidopsis Information Resource (TAIR) BLAST (https://www.arabidopsis.org/Blast/index.jsp) and National Center for Biotechnology Information (NCBI) BLAST (https://blast.ncbi.nlm.nih.gov/Blast.cgi) and were downloaded. The transmembrane domain (TMD) was predicted by TMHMM Server v2.0 (http://www.cbs.dtu.dk/services/TMHMM/). Protein targeting was predicted by TargetP (http://www.cbs.dtu.dk/services/TargetP/). Multiple sequence alignment was carried out using ClustalW2 [28]. Maximum likelihood (ML) phylogenetic analysis of AtPAP2 and its relatives was carried out by MEGA 7.0 software [29]. Bootstrap values at the corresponding nodes were based on 1000 bootstrapping replicates.

### 2.2. Construction of Plasmids

For the localization study of AtPAP2, three constructs of AtPAP2 with different lengths were made with a C-terminal YFP fusion. Full-length, AtPAP2^∆TMD^ or the first 51 amino acids of AtPAP2 (At1g13900) cDNA were PCR-amplified with primer pairs AtPAP2-1/AtPAP2-2, AtPAP2-1/AtPAP2-4, and AtPAP2-1/AtPAP2-3, respectively, from wild type (Columbia, Col) *Arabidopsis* cDNA. The PCR products were digested with restriction endonucleases (New England Biolabs, Ipswish, MA, USA) HindIII/MluI and inserted into binary vector 3302Y4 digested with HindIII/MluI to generate AtPAP2-YFP, AtPAP2^∆TMD^-YFP and SP-YFP, respectively.

To generate signal peptide (SP)-YFP-TMD, two PCR fragments containing the SP-YFP and TMD were amplified using primer pairs AtPAP2-1/YFP-E2 and AtPAP2-6/AtPAP2-3, respectively. The two fragments were digested with HindIII/KpnI and KpnI/MluI, respectively, and cloned into binary vector 3300B digested with HindIII/MluI, generating the plasmid SP-YFP-TMD. For the localization study of YFP-TMD, the TMD sequence of AtPAP2 was PCR-amplified with primer pairs AtPAP2-7/AtPAP2-3. The resulting fragment was digested with NcoI/MluI and cloned into the binary vector 3302NY digested with NcoI/MluI to obtain the plasmid YFP-TMD. Primers used in this study are synthesized by Tsingke (Beijing, China) and listed in Table S1. All plasmid vectors used were binary pCAMBIA-derived T-DNA vectors.

### 2.3. Transient Expression and Microscopy

Transient expression in tobacco was performed as previously described [30]. AtPAP2-YFP, AtPAP2^∆TMD^-YFP, SP-YFP, SP-YFP-TMD and YFP-TMD plasmids were transformed into *Agrobacterium tumefaciens* strain C58C01, and then the bacteria were infiltrated into the epidermal cell layers of tobacco (*Nicotiana benthamiana*) leaves for transient expression under a cauliflower mosaic virus (CaMV) 35S promoter.

Microscope analysis was carried out two days after the infiltration. Fluorescent images of YFP and mCherry were captured with a digital camera coupled with a fluorescent microscope (NE910, Nexcope, Ningbo, China). FM4-64 dye (Invitrogen, Eugene, OR, USA) is a lipophilic styryl compound used to label the plasma membrane in a wide variety of studies. For this purpose, a small piece of leaf tissue was incubated with 5 μM FM4-64 for 20 min and protected from light before visualization. mCherry-HDEL was used as an endoplasmic reticulum (ER) marker [31,32]. The N-terminal SP of isovaleryl-CoA dehydrogenase (IVDH) fused to the N-terminus of mCherry was used as a mitochondria marker [33]. Excitation (Ex) and emission (Em) filter settings were: YFP-Ex 460–490 nm, Em 510–550 nm; FM4-64 and mCherry-Ex 540–580 nm, Em > 595 nm. Images analysis was done by using Adobe Photoshop CC software (https://www.adobe.com/products/photoshop.html).

## 3. Results and Discussion

### 3.1. Bioinformatics Analysis of Purple Acid Phosphatases in Arabidopsis

In order to study the sequence difference between AtPAP2 and other PAPs in *Arabidopsis*, we searched PAP2 homologs and analyzed the TMD and the targeting sequences of individual proteins. As shown in Table 1, almost all of the PAPs were predicted to be targeted to the secretory pathway and secreted out of the cell to extracellular region, such as AtPAP15 [22]. In contrast to the other members in the family, AtPAP2 has a long C-terminus which contains a predicted TMD from 615 aa to 635 aa, and AtPAP9 has a predicted C-terminal TMD from 606 aa to 626 aa. The C-terminal TMD probably functions in membrane localization. This suggests a special role of these two proteins and a close relationship between them.

**Figure 1:**
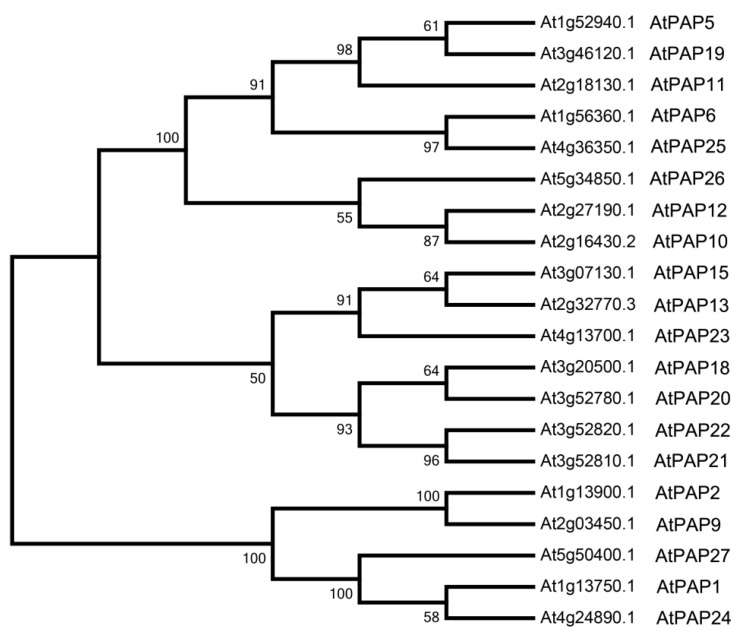
Phylogenetic analysis of AtPAP2 and its homologs in *Arabidopsis*. A phylogenetic tree of the protein sequences of AtPAP2 and their homologs in *Arabidopsis*. Bootstrap values at the corresponding nodes are based on 1000 bootstrapping replicates.

To further study the phylogenetic relationship of AtPAP2 and other PAPs in *Arabidopsis*, a ML phylogenetic tree was constructed using MEGA 7.0. AtPAP2 and AtPAP9, the only two PAP proteins that contain C-terminal TMD in *Arabidopsis* (Table 1), were grouped into the same cluster (Figure 1), suggesting a special evolutionary position and function of these two proteins.

### 3.2. AtPAP2 Is Localized to the Plasma Membrane

**Figure 2:**
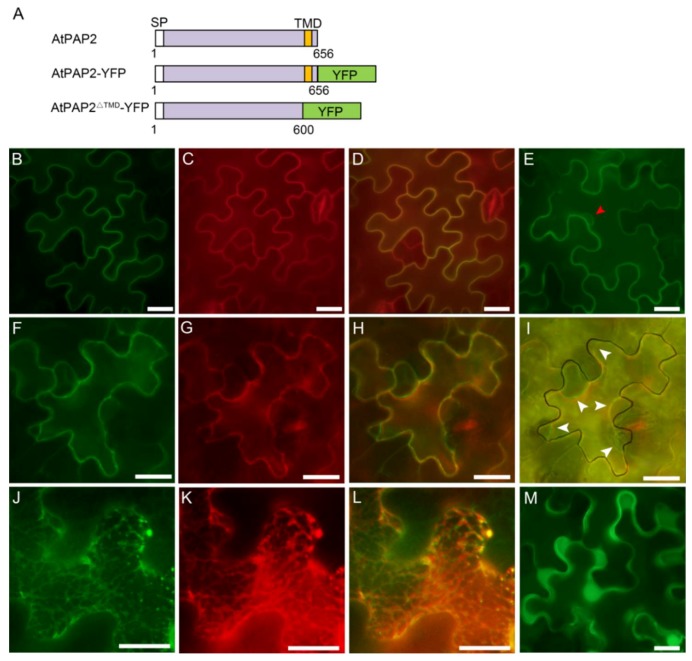
Subcellular localization of AtPAP2-YFP and AtPAP2^∆TMD^-YFP in tobacco epidermal cells. (**A**) Schematic diagram of the AtPAP2-YFP and AtPAP2^∆TMD^-YFP fusion constructs; (**B**–**L**) The full length and the first 600 aa without the TMD of AtPAP2 with a yellow fluorescent protein (YFP) fusion under a cauliflower mosaic virus (CaMV) 35S promoter were used to study the subcellular localization in tobacco leaf epidermal cells by *Agrobacterium*-mediated transient expression. Fluorescence corresponding to the expressed proteins was observed with a fluorescence microscope 48 h after infiltration. (**B**–**I**) AtPAP2-YFP. (**J**–**L**) AtPAP2^∆TMD^-YFP. (**M**) YFP only. (**B**,**E**,**F**,**J**,**M**) YFP. (**C**,**G**) FM4-64. (**K**) mCherry-HDEL. (**D**,**H**,**I**,**L**) Overlay. The red arrow points to the nuclear envelope. (**F**–**I**) The white arrows point to the sites of plasma membrane plasmolysis when treated with 50% sucrose. The border of a cell (**I**) is marked with black color in bright field image. Bar = 10 μm.

It has been shown that AtPAP2 was localized to both chloroplasts and mitochondria in previous studies [24,25], which differ from our bioinformatics analysis. To determine the subcellular localization, AtPAP2 was cloned into a plant transformation vector and fused with a YFP reporter gene (Figure 2A) and then transiently expressed in tobacco (*N. benthamiana*) leaf cells. Two days after the infiltration, the YFP fluorescence signal was observed on the plasma membrane (Figure 2B–E), which was further confirmed by the staining of FM4-64 (Figure 2C), a specific plasma membrane dye and plasmolysis (Figure 2F–I). The results clearly suggested that most of the signals of AtPAP2-YFP were detected on the plasma membrane.

To assess the C-terminal TMD of AtPAP2 for its plasma membrane targeting, we fused 1–600 aa of AtPAP2 without C-terminal TMD to the N-terminus of YFP (AtPAP2^∆TMD^-YFP, Figure 2A) and observed the subcellular localization by transient expression in tobacco leaf cells. The results showed that the YFP fluorescence signal was observed in the cytoplasm, mainly in the ER, which was confirmed by ER marker mCherry-HDEL, while the free YFP was localized to the cytoplasm and nucleus (Figure 2J–M). Overall, these findings indicated that AtPAP2 was localized to the plasma membrane, and the C-terminal TMD is essential for its plasma membrane localization.

### 3.3. AtPAP2 is Targeted to the Plasma Membrane through Secretory Pathway

**Figure 3:**
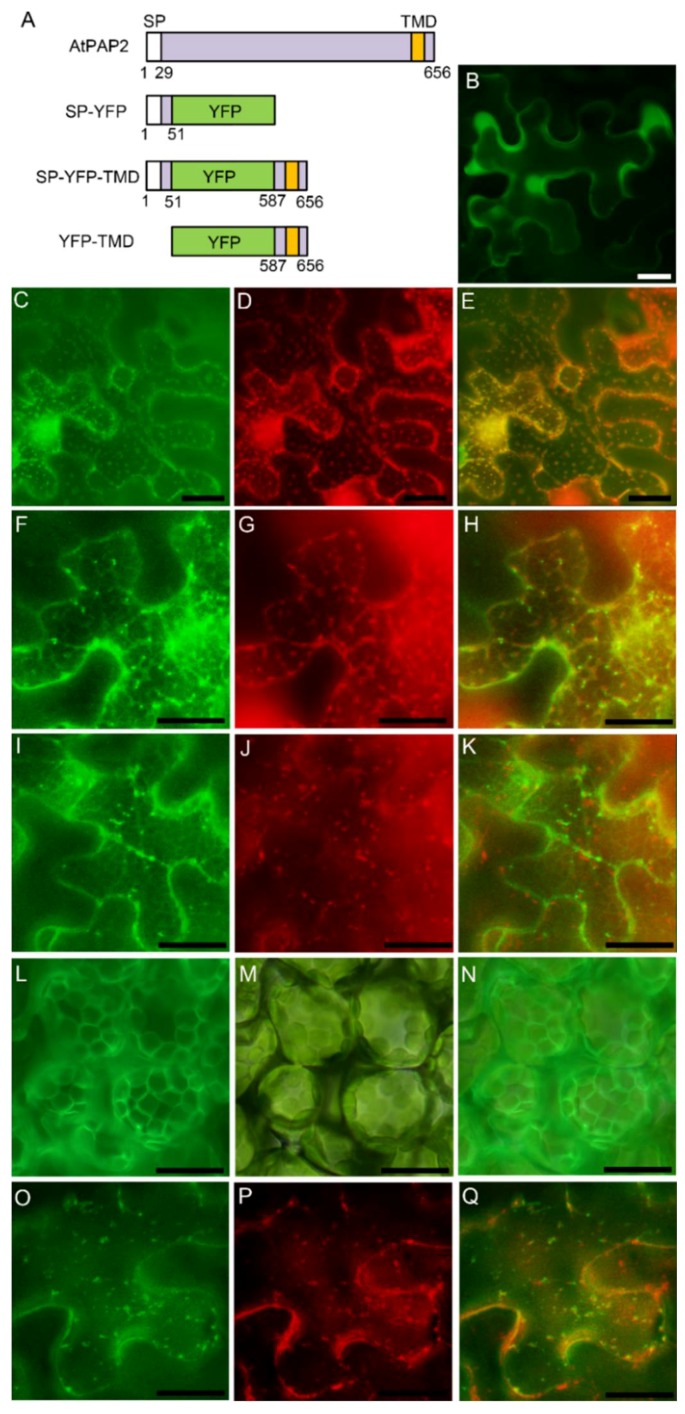
Subcellular localization analyses of SP-YFP, SP-YFP-TMD and YFP-TMD in tobacco leaf cells by *Agrobacterium*-mediated transient expression. (**A**) Schematic diagram of the SP-YFP, SP-YFP-TMD and YFP-TMD constructs. (**B**–**K**,**O**–**Q**) Epidermal cells. (**L**–**N**) Mesophyll cells. (**B**) YFP only. (**C**–**E**) SP-YFP. (**F**–**K**) SP-YFP-TMD. (**L**–**Q**) YFP-TMD. (**B**,**C**,**F**,**I**,**L**,**O**) YFP. (**D**,**G**) mCherry-HDEL (**M**) Bright field. (**J**,**P**) IVDH-SP-mCherry. (**E**,**H**,**K**,**N**,**Q**) Overlay. Bar = 10 μm.

In transient expression, we also observed some weak signals of AtPAP2-YFP on the nuclear envelope (Figure 2E) and in the ER (because the signal is very weak, data will not be shown here), suggesting AtPAP2 may be targeted to the plasma membrane via the secretory pathway. To further investigate the targeting of AtPAP2, the first 51 amino acid residues from the N-terminus including the SP were fused to YFP (Figure 3A) under the control of the CaMV 35S promoter. Fluorescence microscopy analysis showed that the YFP was localized to the ER network (Figure 3C–E), so the SP of AtPAP2 is transported to the plasma membrane via the ER pathway and the subsequent endomembrane system. Thus, it is suggested that AtPAP2 functions in the cell plasma membrane through the secretory pathway.

Previous studies have shown that the C-terminal TMD of AtPAP2 is efficient to target AtPAP2 to the envelope of both chloroplasts and mitochondria by GFP-AtPAP2 [25]. To determine which signal is dominant, the SP or the C-terminal TMD, we constructed SP-YFP-TMD and YFP-TMD and observed their subcellular localization by transient expression in tobacco leaf cells. mCherry-HDEL and IVDH-SP-mCherry were used as ER and mitochondria markers, respectively. The results showed that the signal of SP-YFP-TMD was mainly in the ER (Figure 3F–H), not in the mitochondria (Figure 3I–K), while the signal of YFP-TMD was on the envelope of chloroplasts and mitochondria (Figure 3L–Q). Although TMD itself can lead YFP to the chloroplasts and mitochondria by observing the localization of YFP-TMD in this and previous studies [25], SP is the dominant signal to lead AtPAP2 to the secretory pathway when SP and TMD are simultaneously present in AtPAP2.

Here, we showed that AtPAP2 was targeted to the plasma membrane through the secretory pathway, which is different from previous information. Sun et al. showed AtPAP2 was localized to both the chloroplasts and mitochondria by using GFP-AtPAP2 which will prevent the SP from entering the secretory pathway [24,25]. Although AtPAP2 could be detected in isolated chloroplasts and mitochondria with the AtPAP2 antibody in a previous study [25], plasma membrane contamination in the organelle isolation procedures cannot be ruled out. Nikolovski et al. [34] analyzed the Golgi resident proteins by label-free quantitative mass spectrometry and found that AtPAP2 was a Golgi localized protein, which is in favor of it being involved in the secretory pathway. The correct localization information of AtPAP2 is important for the study of its function.

### 3.4. The Evolutionary Origin of AtPAP2 and AtPAP9

In order to study the origin of AtPAP2 and AtPAP9, we searched PAP2 homologs in other species, predicted the TMD of each protein by TMHMM Server 2.0 and constructed the phylogenetic tree by MEGA 7.0. AtPAP2 homologs can be found in a variety of species, including land plants, green algae, bacteria, fungi and animals (Table S2). PAPs with the C-terminal TMD can only be found in land plants, such as *Physcomitrella patens*, *Oryza sativa*, *Populus trichocarpa* and their ancestor streptophyte green algae (*Klebsormidium nitens*), not in other green algae, bacteria, fungi or animals, thus indicating PAP2 may have a special role in land plants.

**Figure 4:**
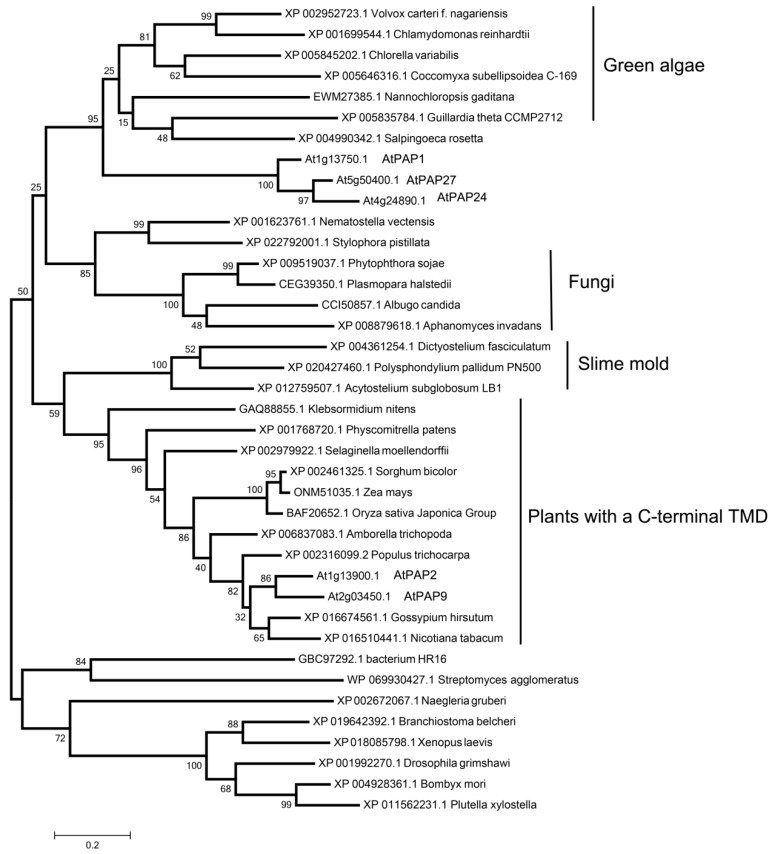
Phylogenetic analysis of AtPAP2 and its homologs in other species. Shown is a phylogenetic tree of AtPAP2, the 4 most related AtPAPs (AtPAP1, AtPAP9, AtPAP24 and AtPAP27), and PAP2 homologs in green algae, fungi, slime mold, and land plants. Bootstrap values at the corresponding nodes are based on 1000 bootstrapping replicates.

Moreover, we compared AtPAP2, its four most related PAPs in *Arabidopsis* (AtPAP1, AtPAP9, AtPAP24 and AtPAP27) and AtPAP2 homologs in other species and constructed the phylogenetic tree for further analysis. As shown in Figure 4, AtPAP2 and AtPAP9 belong to a unique branch of the PAP family with the C-terminal TMD, which have a close relationship with other PAP2-like proteins in streptophyte green algae and slime mold. Green algae PAP2-like proteins have a close relationship to AtPAP1/24/27 (Figure 4). These results clearly showed that PAP2 and other PAPs without C-terminal TMD are in different clusters, indicating their functions may be different. Therefore, AtPAP2 may have evolved from gene duplication during plant landing.

The C-terminal TMD of AtPAP2 is essential for its plasma membrane localization which is conserved across land plants, indicating it may play an important role in the early freshwater adaptation and landing of plants. The mycelium of fungi, protonema of moss and ferns, root hairs and pollen tube of land plants are typical examples of cell polarity. AtPAP2 is highly expressed in the root, stem and pollen of *Arabidopsis*, which require fast growth [16]. Over-expression of AtPAP2 showed faster plant growth in *Arabidopsis* [24,35], *C. sativa* [26] and potato [27]. Thus, AtPAP2 may function in plant tissues of fast growth or polarity with the enzyme activity of protein phosphatase or phosphatase for inorganic P compounds.

## 4. Conclusions

In this study, we examined the targeting of AtPAP2 and found it is localized to the cell plasma membrane. Phylogenetic analysis showed that AtPAP2 and AtPAP9 [5] are clustered with slime mold and fungi, suggesting it may have had a unique role during early plant landing. The C-terminal TMD of PAP2-like proteins is conserved in all land plants. The fast growing and high yield phenotypes of *AtPAP2* overexpressors make *PAP2* a potential gene for crop improvement, but the susceptibility to pathogen infection should be of concern because *AtPAP2* overexpressors were more susceptible to *Pseudomonas syringae* [7]. Our results clarified the localization of AtPAP2 and provide useful information for the future study of AtPAP2 in understanding its special function in crop improvement.

## Figures and Tables

**Table 1:** A bioinformatics analysis of the targeting of purple acid phosphatases (PAPs) in *Arabidopsis.*

Gene Name	AGI ^1^ Code	Protein Length	No. and Position of Transmembrane Domain (TMD)	Subcellular Localization ^2^
*AtPAP1*	At1g13750.1	613 aa ^3^	- ^4^	Secretory Pathway
*AtPAP2*	At1g13900.1	656 aa	1: 615–635 aa	Secretory Pathway
*AtPAP5*	At1g52940.1	396 aa	-	Other
*AtPAP6*	At1g56360.1	466 aa	-	Secretory Pathway
*AtPAP9*	At2g03450.1	651 aa	1: 606–626 aa	Secretory Pathway
*AtPAP10*	At2g16430.1	348 aa	-	Mitochondrion
*AtPAP10*	At2g16430.2	468 aa	-	Secretory Pathway
*AtPAP11*	At2g18130.1	441 aa	-	Secretory Pathway
*AtPAP12*	At2g27190.1	469 aa	-	Secretory Pathway
*AtPAP13*	At2g32770.1	516 aa	-	Secretory Pathway
*AtPAP13*	At2g32770.2	428 aa	-	Other
*AtPAP13*	At2g32770.3	545 aa	-	Secretory Pathway
*AtPAP15*	At3g07130.1	532 aa	-	Secretory Pathway
*AtPAP18*	At3g20500.1	437 aa	-	Secretory Pathway
*AtPAP19*	At3g46120.1	388 aa	-	Secretory Pathway
*AtPAP20*	At3g52780.1	427 aa	-	Secretory Pathway
*AtPAP20*	At3g52780.2	361 aa	-	Secretory Pathway
*AtPAP21*	At3g52810.1	437 aa	-	Secretory Pathway
*AtPAP22*	At3g52820.1	434 aa	-	Secretory Pathway
*AtPAP23*	At4g13700.1	458 aa	-	Secretory Pathway
*AtPAP24*	At4g24890.1	615 aa	-	Secretory Pathway
*AtPAP25*	At4g36350.1	466 aa	-	Secretory Pathway
*AtPAP26*	At5g34850.1	475 aa	-	Secretory Pathway
*AtPAP27*	At5g50400.1	611 aa	-	Secretory Pathway

^1^ AGI: Arabidopsis Genome Initiative; ^2^ The subcellular localization was predicted by TargetP; ^3^ aa, amino acid; ^4^ “-” represents none.

## Supplementary Materials

The following are available online at http://www.mdpi.com/2073-4425/9/5/257/s1. Table S1: Primers used in this study; Table S2: AtPAP2 homologs in various species included in the phylogenetic analysis and the BLAST results.
