# Supplementary material for: AtPAP2, a Unique Member of the PAP Family, Functions in the Plasma Membrane
Source: Genes (Basel). 2018 May 17;9(5):257. doi: 10.3390/genes9050257 (PMC5977197; doi:10.3390/genes9050257)
Supplement: Supplementary file 1 [file genes-09-00257-s001.zip › Table S1.docx]

**Table S1.** Primers used in this study.

| **Name** | **Sequence (5′-3′)** | **Restriction enzymes** |
| --- | --- | --- |
| AtPAP2-1 | CCT AAG CTT TCC TCC GTC GCC ATG ATC | HindIII |
| AtPAP2-2 | CCT ACG CGT TGT CTC CTC GTT CTT GAC | MluI |
| AtPAP2-3 | CCT ACG CGT TGA CGG AGA ATC GAC ACC | MluI |
| YFP-E2 | GGT GGT ACC GTA CAG CTC GTC CAT GCC G | KpnI |
| AtPAP2-5 | CCT ACGCGT TGC TCC TTT GGC ATA CCA C | MluI |
| AtPAP2-6 | CCT GGT ACC AGC AAA GAG AGT ACT AAA ATC CC | Kpn1 |
| AtPAP2-7 | CCT CCA TGG TGA GCA AAG AGA GTA CTA AAA TCC | Ncol |

Restriction sites are underlined.
