# Supplementary material for: AtPAP2, a Unique Member of the PAP Family, Functions in the Plasma Membrane
Source: Genes (Basel). 2018 May 17;9(5):257. doi: 10.3390/genes9050257 (PMC5977197; doi:10.3390/genes9050257)
Supplement: Supplementary file 1 [file genes-09-00257-s001.zip › Table S2.docx]

**Table S2.** AtPAP2 homologs in various species included in the phylogenetic analysis and the BLAST results.

| **Species** | **GenBank accession No.** | **Length**  **(aa)** | **Score** | **Expect** | **Identities** |
| --- | --- | --- | --- | --- | --- |
| *Arabidopsis thaliana* | AT1G13900.1 | 656 | 1367 bits (3538) | 0.0 | 100% |
| *Arabidopsis thaliana* | At2g03450.1 | 651 | 1015 bits (2625) | 0.0 | 72% |
| *Arabidopsis thaliana* | At1g13750.1 | 613 | 221 bits (564) | 9e-58 | 30% |
| *Arabidopsis thaliana* | At5g50400.1 | 611 | 212 bits (540) | 7e-55 | 29% |
| *Arabidopsis thaliana* | At4g24890.1 | 615 | 201 bits (510) | 2e-51 | 27% |
| *Populus trichocarpa* | XP_002316099.2 | 647 | 907 bits (2344) | 0.0 | 68% |
| *Gossypium hirsutum* | XP_016674561.1 | 655 | 882 bits (2279) | 0.0 | 65% |
| *Nicotiana tabacum* | XP_016510441.1 | 652 | 862 bits (2228) | 0.0 | 64% |
| *Sorghum bicolor* | XP_002461325.1 | 653 | 769 bits (1986) | 0.0 | 59% |
| *Oryza sativa Japonica Group* | BAF20652.1 | 676 | 767 bits(1980) | 0.0 | 58% |
| *Zea mays* | ONM51035.1 | 654 | 759 bits(1959) | 0.0 | 58% |
| *Amborella trichopoda* | XP_006837083.1 | 651 | 754 bits (1948) | 0.0 | 61% |
| *Selaginella moellendorffii* | XP_002979922.1 | 646 | 673 bits(1737) | 0.0 | 53% |
| *Physcomitrella patens* | XP_001768720.1 | 668 | 602 bits (1553) | 0.0 | 47% |
| *Klebsormidium nitens* | GAQ88855.1 | 662 | 539 bits (1389) | 0.0 | 44% |
| *Guillardia theta CCMP2712* | XP_005835784.1 | 546 | 225 bits (574) | 9e-66 | 32% |
| *bacterium HR16* | GBC97292.1 | 391 | 123 bits (308) | 2e-31 | 26% |
| *Streptomyces agglomeratus* | WP_069930427.1 | 509 | 86.7 bits (213) | 4e-18 | 29% |
| *Dictyostelium fasciculatum* | XP_004361254.1 | 591 | 398 bits (1023) | 2e-131 | 39% |
| *Acytostelium subglobosum LB1* | XP_012759507.1 | 602 | 385 bits (988) | 4e-126 | 37% |
| *Polysphondylium pallidum PN500* | XP_020427460.1 | 582 | 381 bits (978) | 7e-125 | 36% |
| *Nematostella vectensis* | XP_001623761.1 | 529 | 304 bits (779) | 5e-96 | 34% |
| *Stylophora pistillata* | XP_022792001.1 | 592 | 305 bits (781) | 7e-95 | 34% |
| *Phytophthora sojae* | XP_009519037.1 | 612 | 289 bits (739) | 5e-89 | 34% |
| *Salpingoeca rosetta* | XP_004990342.1 | 569 | 270 bits (689) | 3e-82 | 33% |
| *Plasmopara halstedii* | CEG39350.1 | 675 | 271 bits (692) | 8e-82 | 32% |
| *Albugo candida* | CCI50857.1 | 566 | 258 bits (659) | 5e-78 | 31% |
| *Volvox carteri f. nagariensis* | XP_002952723.1 | 670 | 228 bits (580) | 1e-65 | 31% |
| *Chlorella variabilis* | XP_005845202.1 | 609 | 219 bits (557) | 3e-63 | 30% |
| *Nannochloropsis gaditana* | EWM27385.1 | 754 | 219 bits (559) | 2e-62 | 31% |
| *Coccomyxa subellipsoidea C-169* | XP_005646316.1 | 724 | 209 bits(533) | 5e-59 | 30% |
| *Chlamydomonas reinhardtii* | XP_001699544.1 | 695 | 196 bits (498) | 9e-54 | 33% |
| *Aphanomyces invadans* | XP_008879618.1 | 490 | 176 bits (446) | 3e-48 | 30% |
| *Naegleria gruberi* | XP_002672067.1 | 418 | 157 bits (396) | 4e-42 | 29% |
| *Bombyx mori* | XP_004928361.1 | 433 | 157 bits (397) | 6e-42 | 30% |
| *Branchiostoma belcheri* | XP_019642392.1 | 440 | 150 bits (380) | 2e-39 | 29% |
| *Plutella xylostella* | XP_011562231.1 | 430 | 143 bits (360) | 5e-37 | 29% |
| *Drosophila grimshawi* | XP_001992270.1 | 430 | 141 bits (355) | 2e-36 | 29% |
| *Xenopus laevis* | XP_018085798.1 | 429 | 140 bits (353) | 2e-35 | 28% |
